# Translation from Foreign Journals

**Published:** 1857-06

**Authors:** Ch. F. J. Lehlbach

**Affiliations:** Newark, N. J.


*Translations from Foreign Journals.* By CH. F. J. LEHLBACH, M. D., Newark, N. J.

Jules Rochard concludes a treatise "*On the influence of sea voyages and the residence in warm countries upon the course of pulmonary phthisis*" by the following propositions: 1. Sea voyages hasten the development of pulmonary tuberculosis much more frequently than the contrary. 2. Pulmonary tuberculosis is more frequent in the navy, than in the army. 3. With rare exceptions, phthisis progresses more rapidly on shipboard than on land. 4. Young men, predisposed to tuberculosis, should not be admitted into the navy. 5. In tuberculous persons sea voyages are only advantageous, if they live on board under *special dietetic* relations, and if they are enabled to change climate and locality according to the seasons of the year and atmospheric conditions; all this is easier accomplished on land. 6. Hot countries generally exercise a deleterious influence upon the course of tuberculosis, and accelerate it. 7. This is especially true of tropical coun ries. 8. Even warm countries beyond the tropics are injurious to the majority of tuberculous persons. But few localities are exceptions to this rule, by virtue of their local relations. Residence in such localities protects the patient from acute affections of the respiratory organs, which is a great desideratum in the first stage of phthisis.—*Gaz. Méd. de Paris, Medic. Neuigk.*

*Bromine and bromate of potash*, in the treatment of pseudo-membranous affections, is highly spoken of in the *Journal des Connaiss Méd.*, 1856. A series of cases is adduced which we give in a short extract: 1. A child, aged five years, suffered from a severe angina; cauterization with nitrate of silver and hydrochloric acid proved insufficient; on the 16th day croup symptoms were added; under the use of bromine the child recovered in six days. 2. A young man æt. 26, was attacked with malignant angina; argenti nitrici had been used but without success; several paroxysms of suffocation had already occurred. On the 5th day of his sickness bromine was used, a marked amelioration took place on the next day, and on the 14th day he was reconvalescent. 3. A girl, æt. 9 years, was treated with bromine on the second day of an attack of angina; on the fifth day she was reconvalescent. 4. A woman, æt. 30

years, who after confinement was taken sick with puerperal mania and angina, was very rapidly restored by bromine. 5. A case of angino, in a man, aged 32, complicated with gangrene of the mucous membrane of the mouth, terminated in recovery under the use of bromine. 6. The same result was obtained in a young woman æt. 21, who was attacked with scarlet fever and gangrenous sore throat. 7. Similar favorable results were obtained in several cases of croup.—*Medic. Neuigk.*

*The Effects of Cafeïn upon the Animal System.*—Dr. *Stuhlmann*, of Friedewald, has performed many experiments with this substance upon animals, and comes to the following conclusions: Cafeïn is a *poison* and not a nutrient, as Liebig has asserted. 2. Cafeïn applied to such parts of the body, most proper, produces the death of various animals in relatively small doses. 3. This substance acts as a poison, not by decomposing the blood, but by causing paralysis, when it comes in contact with the nervous system. 4. The symptoms and phenomena, which cafeïn produces in animals, differ according to the dose. The mode of its administration or application, and the impressibility of the animals experimented upon. (We expect shortly to hear one of the moralizing, anathematizing, fulminating and reverberating sermons of Dr. Dixon's "scalpel" on this subject. Meanwhile, we shall continue to take our regular sip.—*Transl.*)—*Ibid.*

*Sudden Death from Rupture of the Spleen.*—A case of this kind (one of the rarities inmedical practice) occurring during a paroxysm of intermittent fever, is related by Dr. *Roser* in the *Wiener Medic Wochensch.* "J. B., aged 42 years, a well digger, strong built, never sick since childhood, had been attacked for the first time two years ago with intermittent fever, which did not yield to the usual remedy, pepper in brandy, which arrested the disease. The cold stages in these attacks are said to have been exceedingly violent, the aid of several menbeing sometimes required to prevent his falling out of bed. Toward the end of September, 1856, after he had been occupied for some time in cleaning a deep pond in a marshy district, he was again seized with the intermittent, accompanied by vomiting, etc., and violent stretching pains in the left side. The cold stage generally lasted from 6–8 hours. On the 25th of the next month (October), during a violent paroxysm, he suddenly tumbled out of bed, uttered a shriek, sustained himself for a few moments upon his feet, then sank on the floor and expired. I found marked pallor and coldness of the whole body, and enormous distension of the abdomen. Only upon the most urgent requests, I was permitted to open the abdominal cavity, 36 hours after death. On opening the abdomen, a mass of dark, thickish blood welled forth, which amounted probably to 4–5 pounds. The spleen was ruptured almost in its middle, and the lower portion hanging down somewhat, though not completely separated. After being washed off, it weighed 1 pound 12½ ounces; its length was 9¾, its breadth 4½ inches;" its thickness could not be exactly determined on account of softening. The liver and the rest of the abdominal organs, were, strange to say, normal, the gall-bladder empty. Examination of thorax and brain was not permitted.—*Medic. Neuigk.*

In connection with this case the translator thinks it not out of place to mention *A Case of Pernicious Intermittent Fever.*—On April, 1856, I made in conjunction with Dr. G. S. Ward, of this city, a *post-mortem* examination of the body of Ellen Gilroy, aged 5 years. Up to August, 1855, she had been a very healthy child. At that time her parents removed into a very malarious portion of one of our suburbs, where they have lived since. About 3–5 months ago Ellen had suffered from an active attack of intermittent. Her mother procured her some quinine, which broke up the fever. On Sunday, April 13th, she was again seized with a chill and subsequent fever. On Monday she was well. Tuesday morning at about 9½ o'clock, after she had been playing with other children in the street, she was again seized with a chill and went to bed. After a little while (20-30 minutes) her mother, engaged about the room, heard a feeble moan, and found her lying on the face. In a few moments the child, apparently unconscious, expired.

*Obduction,* 6 hours P. M; marble-whiteness over the face and body, lips livid; no suffusion of eyes or face, no foam at the mouth or nose. *Abdomen:* great engorgement of all abdominal viscera, especially the *spleen* and liver. The former weighed 14 ounces, its longitudinal diameter was 6½ inches; transversely it measured 4 inches, was rather more friable than usual. No structural change of any organ could be observed. *Thoracic cavity:* Organs normal. *Brain:* examination not permitted. —*Medical & Surgical Reporter.*

